# Supplementary material for: Corporate digital transformation and carbon emission intensity: Empirical evidence from listed companies in China
Source: PLoS One. 2024 Dec 19;19(12):e0313870. doi: 10.1371/journal.pone.0313870 (PMC11658603; doi:10.1371/journal.pone.0313870)
Supplement: Attachment — Submitted filename: Response to Reviewers.docx [file pone.0313870.s002.docx]

**Response to the editor and reviewers**

Dear editor and reviewers,

We are very grateful for your constructive comments and suggestions for our manuscript entitled “The Direct Role, Mechanism Analysis, and Driving Effect of Digital Empowerment of Carbon Reduction in Enterprises ——Empirical evidence from listed companies”(ID: PONE-D-23-41742). we have considered the comments very carefully and have revised the paper accordingly. These comments are all valuable and helpful for improving our article. We have extensively modified our manuscript according to the editor and reviewers’ comments. We hope that the corrections are satisfactory.

In an effort to enhance the characterization of carbon emission performance among listed companies, the authors engaged in detailed discussions with their co-authors and ultimately opted to replace the explanatory variable in their paper from carbon emissions to carbon intensity. The rationale for this decision is outlined below:

①The achievement of 'dual carbon' involves significant economic and social system transformations, necessitating comprehensive changes in both production and lifestyle. This initiative is characterized by its systemic nature, requiring a multi-dimensional, three-dimensional, and complex approach. It goes beyond mere carbon reduction to encompass a holistic and integrated project.

②The current focus of Chinese government policies is on promoting the adoption of green and low-carbon technologies by enterprises in order to shift away from mass production, consumption, and emission patterns. The goal is to establish a green, low-carbon, and recycling economic system, as well as a clean, low-carbon, safe, and efficient energy system. As a result, absolute 'carbon reduction and emission reduction' is not the primary policy orientation at present. Additionally, with the target node for 'carbon peak' set for 2030 and current carbon emissions yet to reach this peak, there is a lack of significant voluntary efforts by enterprises towards achieving absolute carbon reduction.

③Carbon intensity, in comparison to total carbon emissions, is a measure of the carbon emissions generated by an enterprise per unit of economic activity. It serves as a way to evaluate the carbon efficiency of an enterprise during economic expansion, indicating whether the enterprise has successfully managed to limit carbon emissions while expanding its output. Lowering carbon emission intensity necessitates enterprises to decrease carbon emissions while either sustaining or enhancing their primary business revenue. This encourages enterprises to proactively embrace new technologies, procedures, and materials to enhance energy efficiency and diminish carbon emissions.

④The carbon intensity indicator considers the influence of economic growth, allowing businesses to strive for economic development while also considering the reduction of carbon emissions. This approach facilitates a mutually beneficial relationship between economic growth and environmental protection. Solely focusing on carbon emissions can only indicate the total emissions of a business, without directly revealing the efficiency of its economic operations and environmental impact. For businesses emitting the same amount of carbon, those with higher main business income may demonstrate greater carbon emission efficiency.

After careful consideration of the aforementioned factors, we have made the decision to substitute the explanatory variable of carbon emissions with carbon emission intensity in order to improve the scientific and practical value of the research presented in this paper. While the remainder of the paper remains unaltered, additional components such as a framework diagram in the theoretical analysis section, multiple covariance tests, mediation effect tests, the U-test, the description of instrumental variables, and the test of the coefficient of variation between groups have been included based on feedback from two reviewers. We have enlisted the assistance of our native English-speaking colleagues to refine the article, and have subsequently made revisions in alignment with the stylistic guidelines of PLOS ONE.

Point-by-point answers to the kind editor and two kind reviewers are listed below this letter. The reviewer comments are in italic font below, and specific concerns have been numbered. Our response is given in regular font, and changes/additions to the manuscript are shown in blue text.

We have tried our best to make all the revisions clear, and we hope that the revised manuscript can satisfy the requirements for publication. We appreciate for editors and reviewers’ warm work earnestly, and hope that the correction will meet with approval. Thanks again for your consideration of publishing our manuscript in your journal.

We are looking forward to hearing from you soon.

Kind regards,

Kejia Guo, Ph.D

gkjschoolemail@163.com

**Response to the comments of Reviewer #1**

Thank you for your valuable suggestions and comments. We have carefully reviewed and made the necessary corrections based on your feedback. Our revised instructions are as follows:

**1. *In the theoretical analysis section, it is recommended to add a theoretical model framework diagram to facilitate readers to understand the author's research ideas.***

Response1: Thank you for your kind suggestion, we have added the following theoretical model framework diagram to the theoretical analysis section of the article（Lines 321-323）.

**Figure 1. Theoretical framework diagram of the impact of enterprise digital transformation on carbon intensity.**

**2. *It is recommended that the author compare the obtained results with research results in relevant fields and analyze the applicable scenarios of relevant theories based on similarities and differences.***

Response2: Thank you very much for your suggestion, according with your advise, we have added a comparative analysis with the relevant results in the existing field in the conclusion part of the study, which is modified as follows（Lines 756-801）:

(1) At this stage, the digital transformation of listed companies can effectively reduce their carbon emission intensity, and this conclusion is reaffirmed following an endogeneity test and robustness tests. This conclusion aligns with the findings of Zhong and Ma (2022) [12] and Ma and Yang (2023) [63], which suggest that the digital transformation of listed companies yields dual advantages of enhanced main business revenue and reduced carbon emissions. Further, this paper explores the nonlinear impact between enterprise digital transformation and carbon intensity dynamically, and finds that there is a U-shaped relationship between digital transformation and enterprise carbon intensity that decreases and subsequently rises. This pattern echoes the findings of Xiao (2023) [19], who first examined the nonlinear relationship between digitization level and enterprise carbon performance based on a sample of the provincial level in China. this paper further extends the correlation analysis using a more micro-specific sample of firms. This paper further extends the correlation analysis using a more micro-specific sample of firms.

(2) In contrast to the existing studies that focuses on technological progress [64], structural optimization [53], energy use efficiency [63], etc., this paper offers a unique perspective on the digital transformation of listed companies, analyzing it through the lens of 'input-output' dynamics. The study concludes that such transformation can decrease the carbon intensity of listed companies by lowering environmental governance costs and driving green technological innovation. Through a systematic analysis, the paper establishes a connection between digital transformation and reduced carbon intensity, supporting the idea of a U-shaped relationship. This research provides valuable empirical evidence to encourage further digital transformation in listed companies and enhance the environmental benefits of digitalization.

(3) Although existing studies have explored the network and group effects of digital transformation of listed companies through various lenses the supply chain [55], the peer effect [57] and other fields. However, these studies have not further explored whether the digital transformation facilitates the network and group effects of energy conservation and emission reduction through the supply chain and the peer effect. Moreover, there is insufficient focus on the potential of listed companies, as large-scale enterprises in their regions, to assume the social responsibility for energy conservation and emission reduction. Therefore, this paper focuses on the energy-saving and emission reduction effect of digital transformation within the networks and groups. Our findings indicate that the digital transformation of enterprises can significantly reduce the carbon emission intensity of upstream and downstream enterprises and enterprises in the same industry and the same region, through the industrial linkage effect and the cohort effect. This introduces a new dimension of analysis as well as empirical evidence of the carbon reduction effect of listed companies' digital transformation.

(4) Heterogeneity analysis finds that stringent environmental regulations enhance the effectiveness of digital transformation in reducing carbon emissions. Digital transformation exerts a more pronounced effect on capital-intensive and technology-intensive firms compared to labor-intensive firms. Comparing the existing literature, Scholars investigated the variability in carbon performance of enterprises' digital transformation based on enterprise type, enterprise size and enterprise geographic location [65]. Building upon the work of Wang and Li (2024) [26], this paper demonstrates that digital transformation plays a more prominent role in technology-intensive and capital-intensive enterprises. Furthermore, it complements the findings of Song et al.(2022) [37], revealing that the positive impact of digitization on green technological innovation is amplified in companies subject to stringent regional environmental regulations, thus digital transformation leads to more significant reductions in carbon intensity.

***3. The issue of collinearity in data needs to be tested.***

Response3: Many thanks to the reviewing experts for raising the issue, which is a flaw in the benchmark regression part of this paper. Inspired by the reviewing experts, we tested the issue of collinearity in data，specifically as follows（Lines 399-403）:

To ensure the accuracy of the estimation results, we examined the multicollinearity among variables prior to conducting the baseline regression. The Variance Inflation Factor (VIF) of each variable is less than 1.41, significantly lower than 10, suggesting minimal multicollinearity. So, it can be basically concluded that there is no serious multicollinearity between the variables, and the regression model estimation can be carried out further.

**4. *Why is there such a significant difference in R2 in Table 2? The R2 of model (1) is 0, model (4) only added a "Regional effect" compared to model (3), but R2 increased from 0.295 to 0.92.***

Response4: Many thanks to the reviewer for raising this issue, and we apologize for the oversight in data output and table creation. Upon reviewing the Stata code, we identified that a cross-multiplication term of individual and time was incorrectly included in the cluster analysis in the previous regression. Additionally, there was an error in the output command which resulted in the original R2 being displayed instead of the adj-R2, leading to a significant increase in R2. As a result of our operational errors, the scientific integrity of the paper's results was compromised. We have rectified these errors, made necessary corrections to the regression results and empirical tables, and carefully retested to ensure accuracy. The original data and regression codes have been provided for verification. The results of the latest benchmark regression are as follows（Tab 2）：

**Table 2. Descriptive statistics of variables**

|  | (1) | (2) | (3) | (4) | (5) | (6) |
| --- | --- | --- | --- | --- | --- | --- |
|  | CEI | CEI | CEI | CEI | CEI | CEI |
| dig | -0.121** | -0.103 | -0.223*** | -0.166*** | -0.186*** | -0.395*** |
|  | [0.047] | [0.066] | [0.071] | [0.051] | [0.062] | [0.134] |
| dig^2^ |  |  |  |  |  | 0.004** |
|  |  |  |  |  |  | [0.002] |
| roa |  | -1.123 | -2.356** | -2.342*** | -2.283*** | -2.281*** |
|  |  | [0.907] | [0.900] | [0.793] | [0.755] | [0.755] |
| state |  | -6.248 | -2.71 | -2.779 | -2.42 | -2.387 |
|  |  | [5.025] | [3.809] | [3.750] | [4.739] | [4.748] |
| bsize |  | 3.789*** | 0.839 | 2.026 | 2.090* | 2.101* |
|  |  | [1.137] | [1.798] | [1.479] | [1.143] | [1.144] |
| dshare |  | 1.246*** | 0.503 | 0.545* | 0.681*** | 0.681*** |
|  |  | [0.254] | [0.317] | [0.289] | [0.234] | [0.236] |
| bm |  | -1.447 | -88.002** | -90.486** | -85.622** | -85.704** |
|  |  | [13.275] | [40.327] | [40.134] | [34.862] | [34.886] |
| dual |  | -4.936* | -4.869* | -1.59 | -1.905 | -1.913 |
|  |  | [2.821] | [2.583] | [2.186] | [1.172] | [1.178] |
| age |  | 0.065 | -16.265 | -14.867 | -19.576 | -19.649 |
|  |  | [0.242] | [9.738] | [8.744] | [12.966] | [13.023] |
| u-test |  |  |  |  |  | 2.03** |
| Year FE | yes | yes | yes | yes | yes | yes |
| Individual FE | no | no | yes | yes | yes | yes |
| City FE | no | no | no | yes | yes | yes |
| Industry FE | no | no | no | no | yes | yes |
| N | 27196 | 27196 | 26821 | 26819 | 26819 | 26819 |
| R^2^ | 0.001 | 0.003 | 0.183 | 0.189 | 0.191 | 0.191 |

Note: ***, ** and * indicate significance level of 1%, 5%and 10%, respectively.

**5*.From the author's theoretical analysis and model design, reducing the cost of environmental governance, improving the competition degree of the industry, and increasing the green innovation are mediating effects in the impact of digital transformation on carbon emissions, but the author did not rigorously test these mediating effects, such as sobel and bootstrap tests*.**

Response5: Many thanks to your suggestion, we drew on the research idea of Qingfeng Cao (2020) and adopted the approach of introducing the cross-multiplier term between the mechanism variables and the core explanatory variables for the mechanism analysis. Although the standard three-step test of mediation effects was not used, the mechanism analysis in this paper requires further testing. Consequently, the Sobel test and bootstrap test were carried out as the reviewers' advice. The specific changes in the text are outlined below（Lines 602-619）:

In order to ensure the credibility of the results in Table 5, this paper takes two routes to re-test: firstly, it tested the Sobel value of the indirect effect reveals the p-value of Sobel's test all passed the statistical test at the 1% level, firmly rejecting the original hypothesis, so it can be assumed that both the environmental governance cost and green technology innovation have significant mediation effect, with mediation effect share is 23% and 22.5%, respectively. Second, following the methodology proposed by preacher and Hayes (2008), the nonparametric percentile Bootstrap method test was conducted with a sampling number of 1000. The 95% confidence intervals (adjusted for bias) for the coefficients of the indirect effects were calculated. The results showed that the Bootstrap test confidence intervals for environmental governance costs and green technology innovation were [-0.790, -0.168] and [-0.698, -0.250] respectively. Both intervals had negative upper and lower limits, indicating a significant mediation effect for both variables.

**Response to the comments of Reviewer #2**

Thank you for taking the time to review the research content of this paper and for your insightful comments and kind suggestions. Our revised instructions are as follows:

***1*. *Abstract: The authors should clearly and systematically present the direct, mediating, driving, and heterogeneous effects of corporate digital transformation on carbon emissions reduction, ensuring coherence from beginning to end.*)**

Response1: Following your suggestions, our research findings in the abstract section are categorized into four key points: direct effect, mediating effect, driving effect, and heterogeneous effect of enterprise digital transformation on carbon emission intensity. The details are as follows（Lines 22-40）:

The findings reveal that: (1) At this stage, digital transformation in listed companies effectively reduces their carbon intensity, but the relationship between the two is not linear; instead, it exhibits a U-shaped trajectory, initially decreasing then increasing. (2) Analysis of mechanism indicates that costs associated with environmental governance and innovations in green technology serve as critical pathways through which corporate digital transformation influences carbon intensity. (3) The analysis of driving effect suggests that the digital transformation significantly curtails the carbon emission intensity of both upstream and downstream enterprises as well as those within the same industry and geographical region, through industrial linkage and the cohort effect. (4) Heterogeneity analysis elucidates that the digital transformation of enterprises in regions with stronger government environmental regulations has a markedly more pronounced effect on reducing the carbon emission intensity. Furthermore, the carbon emission reduction effect of digital transformation is more potent in capital-intensive and technology-intensive enterprises compared to labor-intensive enterprises.

***2. Literature review and research hypothesis: Given the numerous variables and mechanism pathways discussed in the article, it is recommended to include a framework diagram to elucidate the specific impact mechanisms and pathways.***

Response2: Thank you for your kind suggestion, we have added the following theoretical model framework diagram to the theoretical analysis section of the article to visually illustrate specific impact mechanisms and pathways（Lines 321-323）:

**Figure 1. Theoretical framework diagram of the impact of enterprise digital transformation on carbon intensity.**

***3. Data source and description: The financial sector is generally excluded, at least during the sample selection process. Given the unbalanced panel data, it is necessary to indicate the number of listed companies throughout the sample period.***

Response3: Considering the Reviewer’s suggestion, we made the following changes in the data sources and descriptions: （Lines 388-394）

According to the industry code, we excluded sectors like real estate and financial, ST, *ST, PT and other types of companies, the missing data of key indicators are also deleted. Considering the matching problem of the data, we deleted the data of some listed companies that failed to match, and performed a 1% bilateral tail reduction on all continuous variables. Finally, a final sample of 3,296 companies were obtained in 2021, with a total sample size of 27,196.

***4. Benchmark regression: Significantly positive quadratic terms of explanatory variable may not necessarily be a U-shaped relationship, and the U-shaped curve can be further U-tested by referring to the literature " Thinking about U: Theorizing and testing U- and inverted U-shaped relationships in strategy Research* ".**

Response4: According with your advice, we conducted a U-test for the inverted U-shaped conclusion with reference to the article “Thinking about U: Theorizing and testing U- and inverted U-shaped relationships in strategy Research”. The specific modifications are as follows. (Lines 436-450)

Further, the regression results were analyzed according to Haans’ (2016) U-curve test to assess if they meet the criteria for a U-shaped (inverted U-shaped) curve [48]. The results show that the coefficient of the primary term of the digital construction level is significantly negative ( -0.395***), while the secondary term's coefficient is significantly positive (0.004***), which satisfies the first determination condition; the digital construction level in this paper takes the value range of [0,91], and the slope at the left endpoint is negative (k1 = -0.395), and the slope at the right endpoint is positive (k2= -0.395 +2 × 0.004 × 91 = 0.333), satisfying the second condition; after calculation, the inflection point is x1 = - (-0.395)/(2 × 0.004) = 49.375, which falls within the range of values of the enterprise digital transformation and meets the third condition. As a result, this confirms a U-shaped relationship between the digital transformation of enterprises and carbon intensity, which first decreases and then increases, thereby validating Hypothesis 1 (H1).

In addition, the U-test of the U-shaped conclusion of this paper is supported by using Stata software, The results of the U-shape test show that the t-value is 3.86 and the p-value is 0.000, which passes the U-shape test.

**5. *Endogeneity and robustness test: The construction of instrumental variables yields only cross-sectional data, requiring further explanation on how they are treated as panel data. Additionally, addressing the endogeneity problem with double differencing is commendable, but the parallel trend assumption is a prerequisite for its validity. Therefore, it is advisable to conduct and pass the parallel trend test.***

Response5: Many thanks to the experts' comments, we further added instructions on how to treat instrumental variables as panel data in the text: (Lines 465-473)

Firstly, inspired by Huang et al. (2019) [49], we utilized the number of post offices per million people in each prefecture-level city in 1984 is used as an instrumental variable. Considering that the number of post offices per million people in the city in 1984 is a historical variable, which may be difficult to identify during the estimation of the fixed-effects models [50], we draw on the study of Nunn and Qian (2014) [51], by constructing an interaction term between the number of post offices in the city in 1984 (related to individual changes) and the more exogenous national Internet penetration rate (related to time), is constructed as the instrumental variable (IV1) in this paper and empirically tested using two-stage least squares estimation.

In addition, regarding the reviewer's suggestion of parallel trend testing, we have to explain to the expert here accordingly. We draw on the research idea of Liu, Hui et al. (2022) to indicate the policy shocks in this paper according to whether listed companies have undergone digital transformation during the study period. However, when we exclude the samples with a value of 0 for the dummy variable for digital transformation, the value of 1 for the dummy variable covers the study period of 2007-2021, making it unsuitable for the parallel trend test.

**6. *Analysis of the heterogeneity: The regression coefficients of different subgroups are significant and in the same direction, and cannot be simply compared to the size of the coefficients, which needs to be tested by the coefficient of variation between groups*.**

Response6: Thank you for helpful comments, we are very sorry for our negligence of testing for coefficient of variation between groups. we have made correction according to the Reviewer’s comments. (Lines 693-696,706-715,725-726,735-738)

The test results are shown in the following table, after the test found that under the influence of government environmental regulation and enterprise factor intensity differences, there is a significant difference in the impact of enterprise digital transformation on carbon intensity, but the degree of public attention did not pass the between-groups coefficient test, indicating that the increase in democratic concern at this stage does not significantly increase the enthusiasm of listed companies in China to reduce carbon emissions. This might stem from the absence of a robust monitoring mechanism for corporate carbon disclosure, leading to non-standardized and opaque emission reporting by some listed companies, which fails to form effective social supervision and public opinion pressure. Therefore, the public's concern for environmental pollution does not effectively compel enterprises to improve their social responsibility for low-carbon production, resulting in insignificant difference in the impact coefficients between regions with high public attention and regions with low public attention.

**Table 8. Heterogeneity test**

|  | People's attention | | Level of government environmental regulation | | Factor intensity | | |
| --- | --- | --- | --- | --- | --- | --- | --- |
|  | High | Low | High | Low | Capital intensive | skill-intensive | labor-intensive |
|  | (1) | (2) | (3) | (4) | (5) | (6) | (7) |
| dig | -0.048** | -0.037*** | -1.309*** | -0.063 | -0.857** | -0.307 | -0.199** |
|  | [0.020] | [0.009] | [0.351] | [0.047] | [0.387] | [0.206] | [0.074] |
| N | 7857 | 13975 | 8954 | 17574 | 11262 | 10088 | 9244 |
| **Intergroup coefficient test** | **0.27** | | **4.61**** | | **9.15***** | | |
| R^2^ | 0.236 | 0.185 | 0.292 | 0.233 | 0.243 | 0.045 | 0.412 |

Note: ***, ** and * indicate significance level of 1%, 5%and 10%, respectively.

***7. There are some grammar mistakes in this manuscript. Please ask some native English writers to help you improve this paper.***

Response7: Thank you so much for your careful check, we apologize for the grammar mistakes in our manuscript. We tried our best to improve the manuscript and have also involved native English speakers for language corrections. These changes will not influence the content and framework of the paper. And here we did not list the changes but marked in red in the revised paper. We appreciate for Reviewers' warm work earnestly and hope that the correction will meet with approval.
