# Supplementary material for: Corporate digital transformation and carbon emission intensity: Empirical evidence from listed companies in China
Source: PLoS One. 2024 Dec 19;19(12):e0313870. doi: 10.1371/journal.pone.0313870 (PMC11658603; doi:10.1371/journal.pone.0313870)
Supplement: Attachment — Submitted filename: Response to reviewers.docx [file pone.0313870.s003.docx]

**Response to the editor and reviewers**：

Dear editors and reviewers,

Thank you very much for giving us an opportunity to revise our manuscript. We are very grateful for your constructive comments and suggestions for our manuscript entitled “Corporate digital transformation and Carbon Emission Intensity: Empirical evidence from listed companies in China”(ID: PONE-D-23-41742R1). Those comments are all valuable and very helpful for revising and improving our paper. We have considered the comments very carefully and have revised the paper accordingly. Based on the comments of the two reviewers, we mainly revised the introductory and conclusions and implications sections of the article and improved the references format according to the style of the journal formatting specifications and requirements. All revised contents are marked in red in the revised manuscript with track changes.

Point-by-point answers to two kind reviewers are listed below this letter. The reviewer comments are in italic fontbelow, and specific concerns have been numbered. Our response is given in regularfont, and changes/additions to the manuscript are shown in blue text.

We sincerely appreciate the time and effort invested by editors and reviewers in evaluating our manuscript, and hope that the correction will meet with approval. Thanks again for your consideration of publishing our manuscript in your journal.

We are looking forward to hearing from you soon.

Kind regards,

Kejia Guo, Ph.D

gkjschoolemail@163.com

**Response to reviewer #2:**

***1. The introduction section remains insufficient. It is recommended that*** ***after introducing the topic, the section should include a description of the research strategy and clearly indicate the marginal contribution of this paper compared to existing studies.***

Response1: Thank you for your kind suggestion, we believe that this is indeed necessary. Combining the comments of another reviewer, we have added three new paragraphs to the original introduction. The third paragraph is the main research and conclusions of this paper. The fourth paragraph is the marginal contribution of this paper compared with existing studies, and the fifth paragraph is an introduction to the structure of the paper. The specific additions are as follows: (Lines 95-144)

To the aim, we first established a theoretical analytical framework to assess the impact of corporate digital transformation on carbon intensity from three dimensions: direct impact, mechanisms of action, and driving effects. Subsequently, we conducted empirical tests using panel data from China's A-share listed companies spanning 2007 to 2021, employing a two-way fixed effects model. Finally, we analyzed the heterogeneity of corporate digital transformation's effect on carbon intensity, considering the internal and external environments in which these corporations operate. Our findings indicated that the relationship between enterprise digital transformation and carbon intensity exhibits a U-shaped characteristic, initially decreasing before subsequently increasing. Currently, the digital transformation of listed companies effectively reduces their carbon intensity, with environmental governance costs and green technology innovation serving as critical transmission mechanisms. Furthermore, we observed that corporate digital transformation can not only reduce the carbon emission intensity of upstream and downstream enterprises through industrial linkage effects but also promote carbon emission reductions among enterprises within the same industry and region through cohort effects. Heterogeneity analysis revealed that the carbon emission reduction effect of corporate digital transformation is more pronounced in regions with stronger local government environmental regulations and higher capital and technology intensity.

In comparison to existing literature, this study presents its contributions primarily in three aspects. First, while existing research predominantly examines the impact of digitization on carbon emissions at the macro level [6, 7], there is a notable scarcity of relevant studies at the micro-enterprise level. Furthermore, the mechanisms through which digital transformation influences carbon emission intensity remain unclear. This paper addresses this issue by concentrating on the impact and mechanisms of digital transformation on carbon intensity within micro-production domains. It empirically assesses the carbon emission reduction performance associated with enterprise digital transformation using data from listed companies, thereby enriching the understanding of the drivers of enterprise carbon performance and providing new micro-empirical evidence for digitization-enabled green and low-carbon development. Second, existing literature predominantly adopt an aggregate perspective to analyze the carbon emission effect of digital transformation [8], which struggles to capture the interplay between social resource inputs and carbon outputs. Consequently, this study investigates the environmental performance of enterprise digital transformation from the carbon intensity viewpoint, which more accurately reflects the efficiency of energy use in economic activities and the enterprise's ability for carbon reduction. This approach expands the research on the environmental effects of enterprise digitalization. Third, scholars have increasingly focused on the spatial spillover effects of digital transformation on carbon emission reduction [9]. However, there has been limited exploration from the perspective of the supply chain within the industrial chain. This study explores the driving effect of enterprise digital transformation on carbon emission intensity through the lenses of industrial correlation effects and cohort effects, aiming to provide a more scientific and systematic assessment of the carbon performance following enterprise digital transformation.

The remainder of this paper is organized as follows: The second section presents a literature review of pertinent studies regarding the impact of digital transformation on carbon emissions. The third section establishes a theoretical framework that examines the influence of digital transformation on carbon intensity through three dimensions: direct impact, indirect impact, and driving effect, and formulates the research hypotheses of this study. The fourth section details the models, variables, and data employed in the research. The fifth section discusses the empirical results across six dimensions: benchmark regression, endogeneity issues, robustness testing, mechanism testing, driving effect analysis, and heterogeneity analysis. Finally, the sixth section summarizes the findings and outlines the policy implications of the research.

**2.*The conclusion should succinctly summarize the core findings of the study. The comparative analysis of the results can be discussed after the empirical results in the previous section.***

Response2: Thank you very much for your suggestion. In accordance with your advice, we have incorporated the comparative analysis of our findings with the existing literature into the Results and Discussion section (lines 458-460, 490-493, 776-779, and 792-794). Additionally, we have streamlined the core findings of this paper to enhance conciseness. The revised conclusions are presented below (lines 803-822):

The findings are as follows:

(1) At this stage, the digital transformation of listed companies can effectively reduce their carbon emission intensity, and this conclusion is reaffirmed following an endogeneity test and robustness tests. Further, this paper explores the nonlinear impact between enterprise digital transformation and carbon intensity dynamically, and finds that there is a U-shaped relationship between digital transformation and enterprise carbon intensity that decreases and subsequently rises.

(2) Environmental governance costs and green technology innovation are critical mechanisms through which enterprise digital transformation influences carbon intensity. Notably, both the relationship between enterprise digital transformation and environmental governance costs, as well as that between enterprise digital transformation and green technology innovation, exhibit an inverted U-shaped pattern. This observation supports the U-shaped relationship between enterprise digital transformation and carbon intensity.

(3) The driving effect found that the digital transformation of enterprises can significantly reduce the carbon emission intensity of upstream and downstream enterprises and enterprises in the same industry and the same region, through the industrial linkage effect and the cohort effect.

(4) Heterogeneity analyses show that stringent environmental regulations enhance the effectiveness of digital transformation in reducing carbon emissions. Digital transformation has a stronger carbon intensity reduction effect on capital-intensive and technology-intensive firms compared to labor-intensive firms.

**3.*The authors can refer to “U-shaped relationship between digitalization and low-carbon economy: Mediation and spillover effects, JCLP, 2024” to enhance the corresponding policy implications.***

Response3: Thank you very much for your suggestions and recommendations. This article not only assisted us in enhancing the policy implications section but also contributed to the improvement of the conclusion section. With reference to this article, we have made the following changes to the policy implications section (Lines 833-884):

First, local governments should prioritize policy support for the digital transformation of local enterprises. Considering the stage effect of enterprise digital transformation on carbon intensity, the primary objective is to actively facilitate enterprise digital transformation to achieve an optimal threshold. This strategy requires an acceleration of information and digital infrastructure initiatives, including deploying 5G networks, industrial internet and big data centers, to enhance the support framework for enterprise digital transformation and expedite the emergence of a digitally empowered China. Furthermore, it is essential to integrate green principles in the process of enterprise digital transformation. Local governments need to intensify the oversight and evaluation of enterprises in the process of digital transformation, intensify the enforcement of environmental regulations, advocate for advanced production methodologies such as smart manufacturing and green manufacturing via media publicity, policy clarification, and exchanges of experiences. Additionally, it is important to raise the public's concern about environmental pollution, ensure financial and technological support for labor-intensive enterprises undergoing digital transformation, aiming to reduce carbon emissions from digitalization activities and foster a sustainable digital transformation paradigm.

Secondly, it is important to utilize the optimal threshold of enterprise digital transformation as a constraint to leverage the transmission effects of environmental governance costs and green technology innovation. On the one hand, in addressing the potential surge in costs associated with environmental governance during the early stage of digital transformation, the government could mitigate enterprise burdens by offering financial subsidies or tax relief policies, thereby smoothing transition in the initial stage of digital transformation. Establishing a special fund for digital transformation could spur investments in upgrading environmental protection equipment, applying digital technologies and refining management system. On the other hand, this approach advocates the digitalization, intensification and greening of local traditional industries. Enhancing investments in R&D investment in digital and green technologies and fostering enterprise-driven green technology innovations and demonstrations are vital. Through the establishment of green technology innovation incentives, provide R&D financial support, etc., to stimulate the enthusiasm of enterprises in green technology innovation, promote the transformation of technological innovation achievements into practical applications. In addition, it is necessary to focus on the alternative roles of green technological innovation and digital transformation in reducing the intensity of carbon emission, and strategically allocate innovation resources by combining the needs of different stages of digital transformation and green technological innovation, to avoid the waste of resources and duplication of construction.

Thirdly, the central government needs to strengthen its focus on the comprehensive landscape of the industrial chain to facilitate the interconnection and interoperability of digital deployment, while advancing cross-regional digitalization and collaborative development. Given that companies are not isolated entities in the process of digital transformation and environmental economy activities, it is essential to encourage collaborative cooperation between upstream and downstream enterprises within the industrial chain and supply chain. This includes enhancing the integration of digital technology in the industrial chain and supply chain, promoting the establishment of a green supply chain management system, and supporting leading enterprises in spearheading initiatives related to supply chain integration and innovative low-carbon management. Such efforts are crucial for effectively harnessing the industrial synergy effect associated with the carbon emission reduction performance of digital transformation. Additionally, this approach fosters regional digital synergistic development, augments the interaction and exchange of enterprises in the same industry and region. It encourages the establishment of digital resource-sharing platforms across neighboring areas, thereby facilitating a dynamic flow of data elements and fostering in-depth exchanges of digital technologies. Ultimately, this strategy aims to promotes the cohort effect of enterprises' digital transformations and amplify the spatial spillover effects of carbon emission reduction resulting from digital transformation.

***4. Further improvement of the reference format is recommended.***

Response4: Thank you very much for your advice on the normative aspects of writing this paper. We have further refined the reference format to align with the journal's formatting specifications and requirements. The specific changes are detailed below (lines 900-1068):

1.	Nordhaus WD. The Spirit of Green: The Economics of Collisions and Contagions in a Crowded World. Princeton: Princeton University Press; 2021.

4.	Zhao C, Wang W, Li X. How Does Digital Transformation Affect the Total Factor Productivity of Enterprises? Finance & Trade Economics. 2021;42(07):114-129. doi：10.19795/j.cnki.cn11-1166/f.20210705.001

5.	Liu S, Yan J, Zhang S, Lin H. Can Corporate Digital Transformation Promote Input-Output Efficiency? Journal of Management World. 2021;37(05):170-190. doi: 10.19744/j.cnki.11-1235/f.2021.0072

8.	Shang Y, Raza SA, Huo Z, Shahzad U, Zhao X. Does enterprise digital transformation contribute to the carbon emission reduction? Micro-level evidence from China. International Review of Economics and Finance. 2023; 86:1-13.

15.	Liu H, Chen Z, Chen X. A Study on the Environmental Performance of Digital Transformation——Micro Evidence from Chinese Manufacturing Listed Companies. Journal of Social Sciences. 2023(05):126-137. doi: 10.13644/j.cnki.cn31-1112.2023.05.015

18.	Usman A, Ozturk I, Hassan A, Zafar SM, Ullah S. The effect of ICT on energy consumption and economic growth in South Asian economies: An empirical analysis. Telemat Inform. 2021;58:101537. doi: 10.1016/j.tele.2020.101537

21.	Zheng R, Wu G, Cheng Y, Liu H, Wang Y, Wang X. How does digitalization drive carbon emissions? The inverted U-shaped effect in China. Environmental Impact Assessment

Review. 2023;102:107203.

22.	Shobande OA. Decomposing the Persistent and Transitory Effect of Information and Communication Technology on Environmental Impacts Assessment in Africa: Evidence from Mundlak Specification. Sustainability-Basel. 2021;13(9):4683.

45.	Wu J, Xia Q, Li Z. Green innovation and enterprise green total factor productivity at a micro level: A perspective of technical distance. J Clean Prod. 2022;344:131070.

53.	Nunn N, Qian N. US Food Aid and Civil Conflict. Am Econ Rev. 2014;104(6):1630-66.

61.	Liu Y, Zhou S. The Coordination Mechanism of Formal and Informal Environmental Regulation Reducing Pollution and Carbon Emission Benefits. Collected Essays On Finance and Economics. 2023(08):103-112. doi: 10.13762/j.cnki.cjlc.2023.08.001

Special thanks to you for your good comments.

**Response to reviewer #3**

We sincerely thank you for your thoughtful and kind suggestions, which will be invaluable for improving our article and guiding our future submissions. As you suggested, we have uploaded the revised manuscript, highlighting the modified sections.

***1*. *The current introduction is a bit too brief, and it's suggested that the author add three more paragraphs.***

Response1: Based on your suggestion, we have added three new paragraphs to the original introduction, and the contribution in the literature review removed. The third paragraph is the main research content and conclusion of this paper. The fourth paragraph is the marginal contribution of this paper compared with existing studies, and the fifth paragraph is an introduction to the structure of the paper. The specific additions are as follows (Lines 95-144):

To the aim, we first established a theoretical analytical framework to assess the impact of corporate digital transformation on carbon intensity from three dimensions: direct impact, mechanisms of action, and driving effects. Subsequently, we conducted empirical tests using panel data from China's A-share listed companies spanning 2007 to 2021, employing a two-way fixed effects model. Finally, we analyzed the heterogeneity of corporate digital transformation's effect on carbon intensity, considering the internal and external environments in which these corporations operate. Our findings indicated that the relationship between enterprise digital transformation and carbon intensity exhibits a U-shaped characteristic, initially decreasing before subsequently increasing. Currently, the digital transformation of listed companies effectively reduces their carbon intensity, with environmental governance costs and green technology innovation serving as critical transmission mechanisms. Furthermore, we observed that corporate digital transformation can not only reduce the carbon emission intensity of upstream and downstream enterprises through industrial linkage effects but also promote carbon emission reductions among enterprises within the same industry and region through cohort effects. Heterogeneity analysis revealed that the carbon emission reduction effect of corporate digital transformation is more pronounced in regions with stronger local government environmental regulations and higher capital and technology intensity.

In comparison to existing literature, this study presents its contributions primarily in three aspects. First, while existing research predominantly examines the impact of digitization on carbon emissions at the macro level [6, 7], there is a notable scarcity of relevant studies at the micro-enterprise level. Furthermore, the mechanisms through which digital transformation influences carbon emission intensity remain unclear. This paper addresses this issue by concentrating on the impact and mechanisms of digital transformation on carbon intensity within micro-production domains. It empirically assesses the carbon emission reduction performance associated with enterprise digital transformation using data from listed companies, thereby enriching the understanding of the drivers of enterprise carbon performance and providing new micro-empirical evidence for digitization-enabled green and low-carbon development. Second, existing literature predominantly adopt an aggregate perspective to analyze the carbon emission effect of digital transformation [8], which struggles to capture the interplay between social resource inputs and carbon outputs. Consequently, this study investigates the environmental performance of enterprise digital transformation from the carbon intensity viewpoint, which more accurately reflects the efficiency of energy use in economic activities and the enterprise's ability for carbon reduction. This approach expands the research on the environmental effects of enterprise digitalization. Third, scholars have increasingly focused on the spatial spillover effects of digital transformation on carbon emission reduction [9]. However, there has been limited exploration from the perspective of the supply chain within the industrial chain. This study explores the driving effect of enterprise digital transformation on carbon emission intensity through the lenses of industrial correlation effects and cohort effects, aiming to provide a more scientific and systematic assessment of the carbon performance following enterprise digital transformation.

The remainder of this paper is organized as follows: The second section presents a literature review of pertinent studies regarding the impact of digital transformation on carbon emissions. The third section establishes a theoretical framework that examines the influence of digital transformation on carbon intensity through three dimensions: direct impact, indirect impact, and driving effect, and formulates the research hypotheses of this study. The fourth section details the models, variables, and data employed in the research. The fifth section discusses the empirical results across six dimensions: benchmark regression, endogeneity issues, robustness testing, mechanism testing, driving effect analysis, and heterogeneity analysis. Finally, the sixth section summarizes the findings and outlines the policy implications of the research.
